# Application time and persistence of transcranial direct current stimulation (tDCS) against neuronal death resulting from transient cerebral ischemia

**DOI:** 10.1186/s42826-022-00121-8

**Published:** 2022-05-08

**Authors:** Jong-Hun Lee, Bo Hyun Jung, Ki-Yeon Yoo

**Affiliations:** Department of Anatomy, College of Dentistry, Gangneung-Wonju National University, 7, Jukheon-gil, Gangneung, 25427 Korea

**Keywords:** Neuroprotection, Neuronal death, tDCS, Transient cerebral ischemia

## Abstract

**Background:**

Transcranial direct current stimulation (tDCS) has been studied as a tool to stimulate the functional recovery of neurons after stroke. Although this device has recently begun to be utilized for providing neuroprotection in stroke, research on its application conditions is lacking. This study aimed to examine the effects of various tDCS application conditions on cerebral ischemia. Ischemia was induced for 5 min in a gerbil model. The application of tDCS comprised a 20 min stimulation—20 min rest—20 min stimulation protocol, which was implemented simultaneously with the induction of cerebral ischemia. Application time of the tDCS effect on ischemia was confirmed by sampling brain tissues after stimulation using 0.2 mA tDCS at 0, 5, 10 and 60 min after ischemia.

**Results:**

Persistence of the tDCS effect on ischemia was confirmed by sampling brain tissues 5, 7, and 10 days post stimulation, with 0.2 mA tDCS after ischemia. Furthermore, the tissues were stained with cresyl violet and Fluoro-Jade C so as to determine the reduction in neuronal death under all application conditions.

**Conclusions:**

The application of tDCS can be used as a useful intervention for acute phase stroke due to its sustained neuroprotective effect.

## Background

Ischemic stroke induced by cerebral blood flow blockage is a neurological deficit arising from the occlusion of abnormal cerebral blood flow and the consequent damage to the corresponding area [1, 2]. It has a high incidence rate of approximately 85%. During ischemia, oxygen and energy are depleted in neurons [3]. Additionally, oxidative stress, nitrification stress, inflammation, and apoptosis in neurons are known to result from non-homeostatic pathways [4–6].

In previous study have shown that in the stroke mouse model induced by middle cerebral artery (MCA) occlusion, the infarct volume is reduced due to the cathodal tDCS applied intermittently for 40 min [7]. And tDCS was accelerated motor function recovery and induced neurogenesis in ischemia rat model by transient occlusion of MCA [8]. Transcranial direct current stimulation (tDCS) is a non-invasive treatment method that stimulates the brain with direct current electric fields (DCEFs) applied externally, and has been used to regulate brain activity in various neurological diseases [9]. tDCS is to transfer weak direct current by placing the brain between two electrodes, and is classified into anodal tDCS or cathodal tDCS depending which electrode is placed [10].

However, it has been reported that anodal tDCS may not have a stable effect on cortical excitability [11]. Also, in previous study reported that anodal stimulation increased the volume of postischemic lesion and increased blood brain barrier [7]. Therefore, in this study, a safer cathode tDCS was used.

Since studies on applying tDCS to ischemia are deficient, extensive studies on the effects of cathodal tDCS on ischemia should continue. Therefore, the present study utilized an experimental animal model induced with transient cerebral ischemia to study the neuronal protective effect according to the tDCS application time and tDCS persistence.

## Results

### Sham and Isch group

#### Cresyl violet staining (CV staining)

**Fig. 1:**
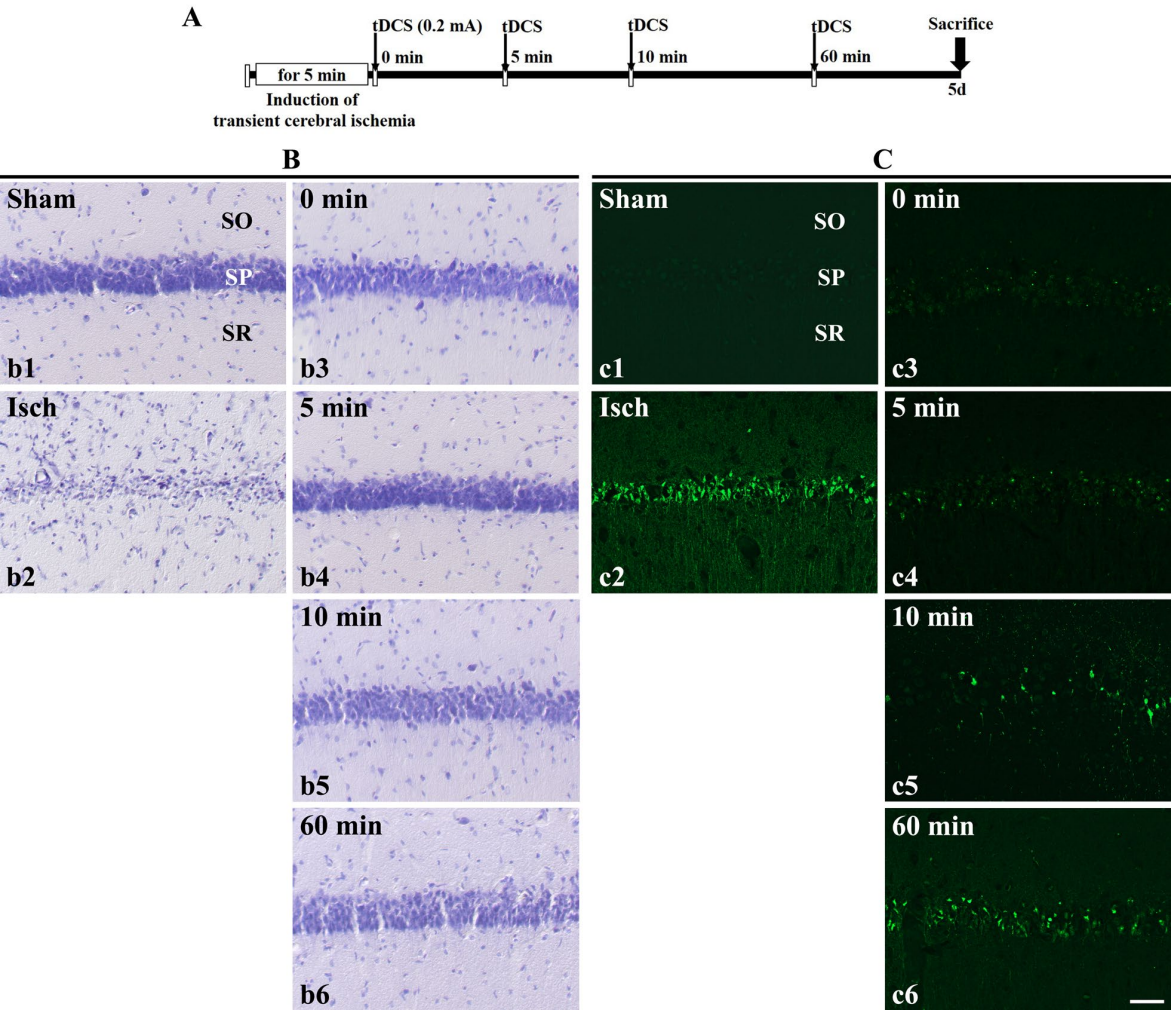
**A** The application of catodal tDCS was performed immediately (0 min), 5 min, 10 min, and 60 min after induction of transient cerebral ischemia for 5 min. The sacrifice point is 5 days after its tDCS application. **B** CV staining and **C** F-J C histofluorescence staining in gerbil hippocampal CA1 regions. (**b1** and **c1**) In the sham group, several CV-positive cells were observed primarily in the SP layer of the hippocampal CA1 regions. However, no F-J C-positive cells were identified. (**b2** and **c2**) In the Isch group, a few CV-positive cells and several F-J C-positive cells were observed. (**b3**–**b6** and **c3**–**c6**) In the 0 min, 5 min, 10 min and 60 min group, CV-positive cells were observed primarily in the SP layer of the hippocampal CA1 regions. A few F-J C-positive cells were observed. SO, stratum oriens; SR, stratum radiatum; SP, stratum pyramidal. Scale bar = 50 µm (n = 7 per group)

**Fig. 2:**
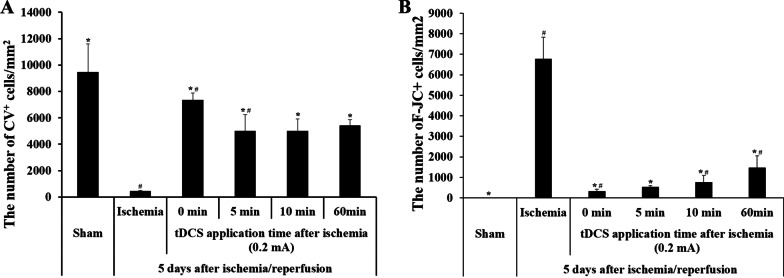
The number of **A** CV- positive cells and **B** F-J C-positive cells corresponding to the application time of cathodal tDCS after the induction of transient cerebral ischemia in various groups (0 min, 5 min, 10 min, and 60 min). (n = 7 per group; *P < 0.05, significantly different from the Isch group; #*P* < 0.05, significantly different from the pre-adjacent group.) The bars indicate the means ± SEM

**Fig. 3:**
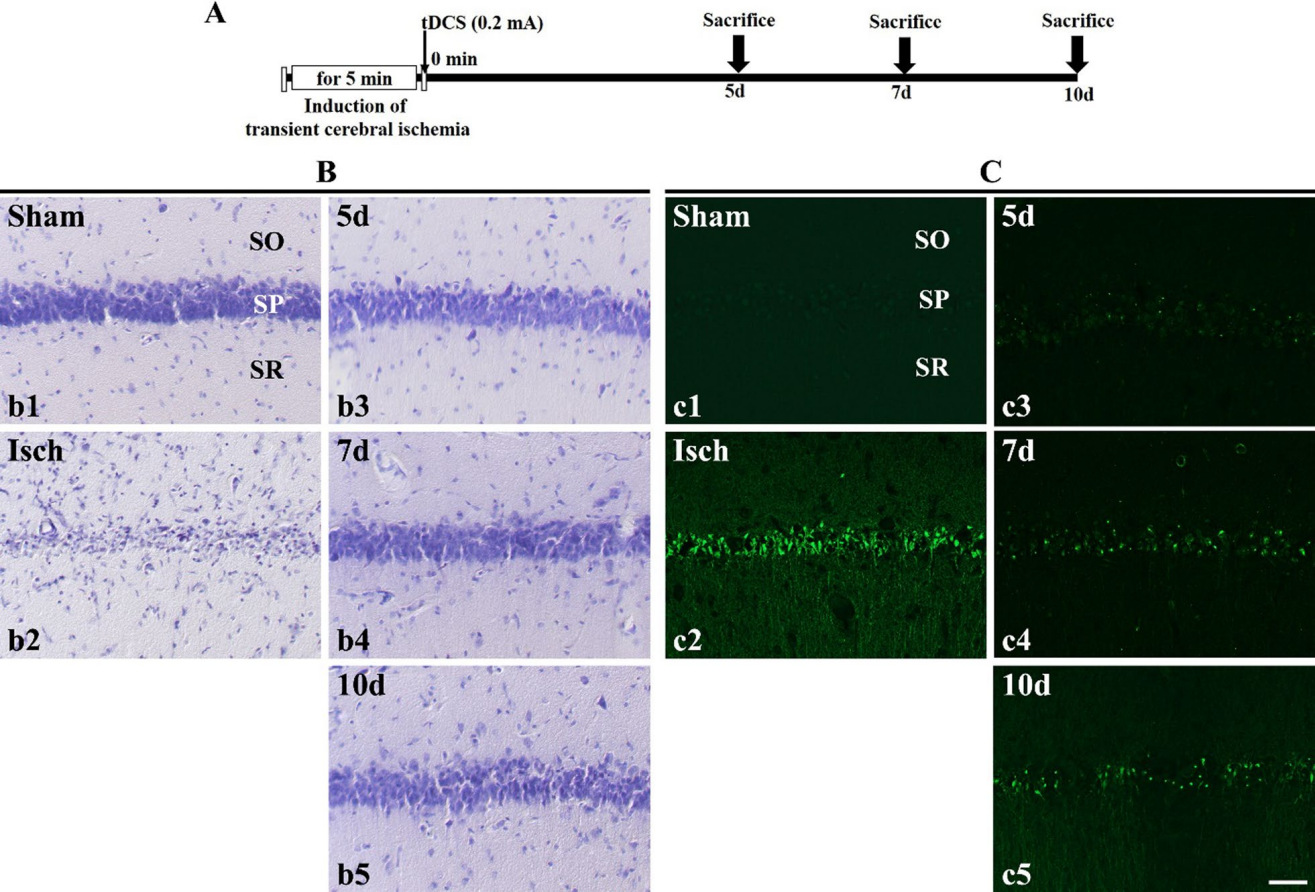
**A** The application of cathodal tDCS was performed immediately after the induction of transient cerebral ischemia. The sacrifice point is 5 days, 7 days, and 10 days after its tDCS application. **B** CV staining and **C** F-J C histofluorescence staining in gerbil hippocampal CA1 regions. (**b1** and **c1**) In the sham group, several CV-positive cells were observed primarily in the SP layer of the hippocampal CA1 regions. However, no F-J C-positive cells were identified. (**b2** and **c2**) In the Isch group, a few CV-positive cells and several F-J C-positive cells were observed. (**b3**–**b5** and **c3**–**c5**) In the 5 days, 7 days and 10 days group, CV-positive cells were observed primarily in the SP layer of the hippocampal CA1 regions. A few F-J C-positive cells were observed. SO, stratum oriens; SR, stratum radiatum; SP, stratum pyramidal. Scale bar = 50 µm. (n = 7 per group)

**Fig. 4:**
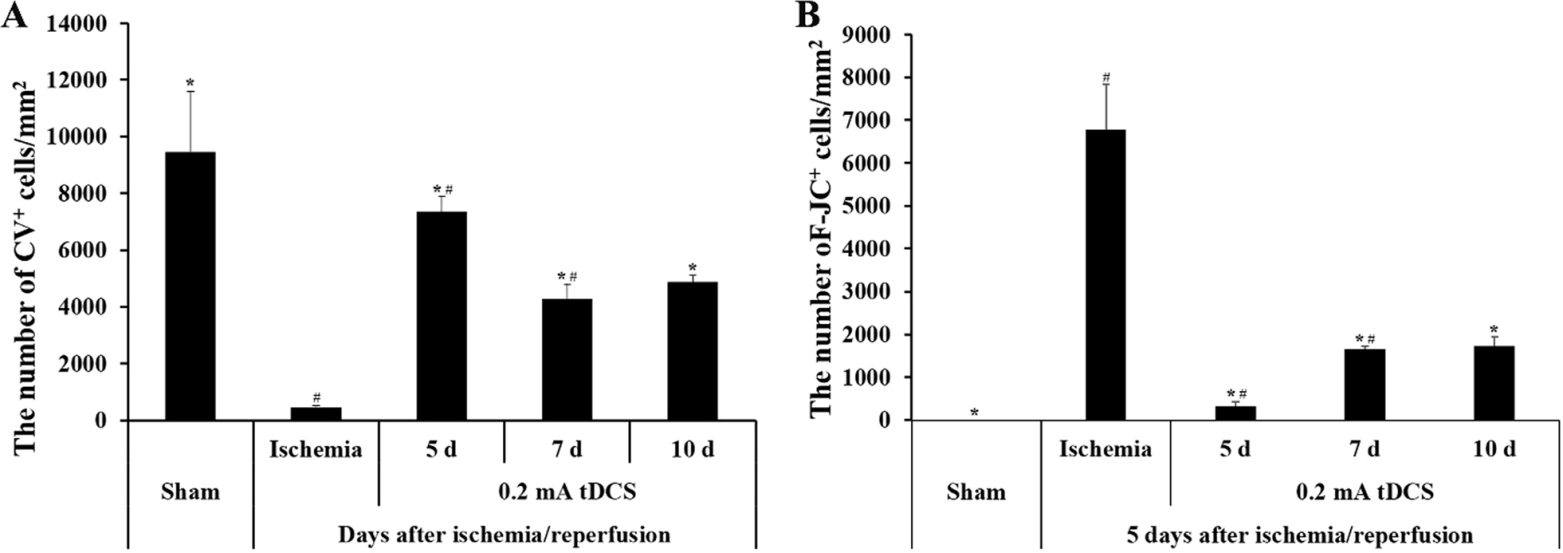
The number of **A** CV- positive cells and **B** F-J C-positive cells with regard to the persistence of cathodal tDCS following the induction of transient cerebral ischemia in various groups (after 5 days, 7 days, and 10 days). (*n* = 7 per group; *P < 0.05, significantly different from the Isch group; ^#^*P* < 0.05, significantly different from the pre-adjacent group.) The bars indicate the means ± SEM

CV -positive cells were observed in all layers of the hippocampal cornu ammonis (CA1) region in the sham group. In particular, they were observed at high densities in the stratum pyramidal layer (Fig. 1Bb1 or Fig. 3Bb1). From the cell count, the number of CV -positive cells in the sham group was identified to be 9058.292 ± 2126.981 cells/mm^2^ (Fig. 2A or Fig. 4A). Contrastingly, CV-positive cells exhibited a decrease in the hippocampal CA1 regions of the Isch group (Fig. 1Bb2 or Fig. 2Bb2), with a cell count of 339.583 ± 54.882 cells/mm^2^ (Fig. 2A or Fig. 4A). Compared to the sham group, the survival rate of neurons was approximately 3.75%, while the number of surviving neurons decreased by approximately 96.25%. These results validated the assumption that neuronal apoptosis was induced in the hippocampal CA1 region owing to transient cerebral ischemia induction.

#### Fluoro-Jade C staining (F-J C staining)

F-J C-positive cells were not observed in any layer of the hippocampal CA1 regions in the sham group (Fig. 1Cc1 or Fig. 3Cc1). However, these cells were observed at high densities in the stratum pyramidal layer of the Isch group (Fig. 1Cc2 or Fig. 3Cc2). From the cell count, the number of F-J C-positive cells in this group was determined to be 6773.497 ± 1062.898 cells/mm^2^ (Fig. 2A or Fig. 4A). Consequently, it was confirmed that neuronal apoptosis was induced in the hippocampal CA1 region due to transient cerebral ischemia induction.

### Experimental groups corresponding to the application time of cathodal tDCS following the induction of transient cerebral ischemia (at 0, 5, 10, and 60 min)

#### CV staining

CV -positive cells were observed in the hippocampal CA1 regions of all groups (Fig. 1Bb3–Bb6). In the 0 min group, the number of CV-positive cells was observed to be 7327.324 ± 542.345 cells/mm^2^ (Fig. 2A). Furthermore, compared to that in the sham group, the survival rate of neurons was approximately 89.89%. Similarly, the 5 min group had a CV -positive cell count of 4793.547 ± 1254.332 cells/mm^2^ (Fig. 2A). The survival rate of neurons in this group was approximately 52.92%, as compared to that in the sham group. In the 10 min group, the concentration of CV positive cells were evaluated as 4823.963 ± 914.325 cells/mm^2^ (Fig. 2A). Compared to that in the sham group, the neurons in this group exhibited a survival rate of approximately 53.25%. Finally, the CV-positive cell count obtained for the 60 min group was 5432.714 ± 427.983 cells/mm^2^ (Fig. 2A). while the survival rate of neurons was approximately 59.98%, when compared to that in the sham group.

#### F-J C staining

F-J C-positive cells were observed in the hippocampal CA1 regions across all the groups (Fig. 1Cc3–Cc6). In the 0 min group, the number of F-J C-positive cells was determined as 323.318 ± 109.293 cells/mm^2^ (Fig. 2B). Additionally, the rate of apoptosis was approximately 4.77%, when compared to that of the Isch group. Similarly, the 5 min group was observed to have an F-J C-positive cell count of 547.683 ± 42.462 cells/mm^2^ (Fig. 2B). Compared to that of the Isch group, the rate of apoptosis in the 5 min group was approximately 8.09%. The 10 min group exhibited F-J C-positive cells at a concentration of 672.487 ± 326.517 cells/mm^2^ (Fig. 2B), along with an apoptotic rate of approximately 9.93%, when compared to that in the Isch group. With regard to the 60 min group, the F-J C-positive cell count was identified to be 1473.121 ± 582.02 cells/mm^2^ (Fig. 2B), while the rate of apoptosis compared to that of the Isch group was approximately 21.75%.

### Experimental groups corresponding to the persistence of cathodal tDCS after induction of transient cerebral ischemia (after 5 days, 7 days, and 10 days)

#### CV staining

To confirm the persistence of cathodal tDCS following the induction of transient cerebral ischemia, the neuronal protective effect was evaluated according to the timing of tissue extraction after cathodal tDCS application. Consequently, CV-positive cells were observed in the hippocampal CA1 regions of all the groups (Fig. 3Bb3-Bb5). After 5 days, the number of CV-positive cells in the respective group was observed to be 7327.324 ± 542.435 cells/mm^2^ (Fig. 4A). Compared to that in the sham group, the survival rate of neurons in this group was approximately 80.89%. Similarly, the CV-positive cell count in the 7 days group was 4193.537 ± 427.983 cells/mm^2^ (Fig. 4A), while the survival rate of neurons was approximately 46.30%, when compared to that in the sham group. In the 10 days group, the number of C-V-positive cells was evaluated as 4963.916 ± 234.872 cells/mm^2^ (Fig. 4A). Additionally, when compared to that in the sham group, the survival rate of neurons was approximately 54.80%.

#### F-J C staining

Apoptosis was examined in accordance with the timing of tissue extraction following the cathodal tDCS application to confirm the persistence of cathodal tDCS after the induction of transient cerebral ischemia. F-J C-positive cells were observed in the hippocampal CA1 regions across all the groups (Fig. 3Cc3–Cc5). In the 5 days group, the F-J C-positive cell count was observed to be 323.318 ± 109.293 cells/mm^2^ (Fig. 4B). Furthermore, compared to that in the Isch group, the rate of apoptosis was approximately 4.779%. Similarly, the 7 days group had an F-J C-positive cell count of 1653.501 ± 71.586 cells/mm^2^ (Fig. 4B), while the rate of apoptosis was approximately 24.40%, when compared to that in the Isch group. Finally, for the after 10 days group, the number of F-J C-positive cells was observed to be 1725.11 ± 229.381 cells/mm^2^ (Fig. 4B). Additionally, compared to that in the Isch group, the rate of apoptosis was approximately 25.45%.

## Discussion

The brain is very densely populated with neurons. Paralysis of the body manifests even if blood flow to the brain ceases for only 20 s, and the neurons begin undergoing apoptosis when blood flow is terminated for 4 min. Consequently, if stroke is treated within a short time after its occurrence, the treatment efficacy is greatly enhanced [12]. Tissue-type plasminogen activator (tPA), a treatment modality for stroke, has a limited time range, making its application arduous. Therefore, it is necessary to develop new treatments and combination therapies for this ailment. To this end, research on the applications of tDCS in this capacity has been an ongoing undertaking. In this study, we attempted to confirm the potential protective effects of cathodal tDCS when applied in neurons after transient cerebral ischemia.

The data obtained in this study indicated that approximately 96.25% of neurons underwent apoptosis in the hippocampal CA1 regions after 5 min of transient cerebral ischemia. These results are consistent with those of neuronal damage in the hippocampal CA1 regions caused by common carotid artery occlusion in gerbils from previous studies [13]. It has been reported that cathodal tDCS can recover brain damage and neurological deficits caused by MCAO in SD rats, since cathodal tDCS inhibits neuroinflammation [14]. In the acute phase of stroke, cathodal stimulation preserved cortical neurons in the brain from ischemic injury and inflammation [7]. In addition, application of tDCS improved motor function and increased dendritic spine density in post-stroke SD rats [15]. When dendritic spine density increases, neural plasticity is promoted. When neural plasticity is promoted, the nervous system's ability to modify itself functionally and structurally increases as neural plasticity is a component that responds to neural development and normal functioning [16]. When cathodal tDCS was applied after the induction of transient cerebral ischemia, it was observed that all groups had a high survival rate of neurons. These results suggest that the application of cathodal tDCS has a neuroprotection effect following transient cerebral ischemia.

Especially, in many treatments study after ischemia, it was emphasized the importance of early treatment. In the results of this study, when cathodal tDCS was applied within a rapidly time after transient cerebral ischemia, the survival rate of neurons was high and the apoptosis rate was low. These results suggest that the rapid application of cathodal tDCS can decrease apoptosis caused by transient cerebral ischemia. This tend to be consistent with the emphasis on early treatment within 60 min after acute ischemic stroke [17]. In previous studies, application of tDCS immediately after focal brain ischemia resulted the amount of cerebral infarction more 10% reduction compared to non-immediately [18]. It has been reported that early NBO (Normobaric hyperoxia) treatment can slow ischemic Blood–brain barrier (BBB) injury and improve the outcome of delayed tPA treatment [19]. When NBP (dl-3n-butylphthalide) was administered within 24 h after MCAO, the infarct volume decrease, and angiogenesis enhance [20]. In addition, as a result of treatment with 5% magnesium sulfate at 2, 6 and 8 h after MCAO, the survival rate was significantly increased only in the at 2 h treatment group [21]. These results mean that in the outbreak of brain injury, treatment should be received as soon as possible. Therefore, these previous studies and the results of this study suggest that the neuroprotective effect more increase as tDCS is applied within a short period of time after transient cerebral ischemia.

In this study, it was confirmed that the application of tDCS immediately after transient cerebral ischemia was effective on neuronal cell death, and to determine how long this effect lasted. There are many studies that have applied tDCS consecutive days, but there is no study on how long one application of tDCS lasts in transient cerebral ischemia gerbil model. In a previous MCAO study using rats, it was reported that the effect of tDCS did not appear on the 3 days of tDCS application [18]. Therefore, the minimum sacrifice day after application was set to 5 days. And in this study, cathodal tDCS was immediately applied after ischemia induction to confirm duration because the protective effect of neurons was more efficacious when tDCS was applied immediately after ischemia induction.

In this study, a high survival rate of neurons combined with a low apoptosis rate were observed in the brain tissue 5 days after the application of cathodal tDCS. These results indicate that the efficacy of cathodal tDCS is persistent for a period of 5 days. Also, neuroprotective effects were also observed on the 7th and 10th days after tDCS application, but showed a significantly decreased with tDCS effective when compared to the 5 days after tDCS application. In addition, since there was no significant difference between the 7 and 10 days, it is suggested that the neuroprotective effect was maintained and decreased from the 7 days after tDCS application. Therefore, it is suggested that applying cathodal tDCS at intervals of at least 5 days is effective in preventing brain function degradation by protecting the neurons.

## Conclusions

In summary, this study suggested that the application of cathodal tDCS after the induction of ischemic stroke exerts a protective effect on neurons in the brain. When cathodal tDCS was applied immediately following an ischemic stroke, the protective effect of neurons was significantly increased. In addition, the application of tDCS at short intervals is more efficacious in providing protection to the neurons, indicating that cathodal tDCS can safeguard against brain damage caused by ischemia. These results suggest that tDCS can be used as a useful intervention for the treatment of transient cerebral ischemia.

## Methods

### Experimental animal

Male Mongolian gerbils (6-months-old, 75–85 g of body weight) purchased from Orient Bio (Seongnam, Korea) were used in this study. The animals were maintained at 22 °C and 50% humidity, and were fed freely every 12 h of the day/night cycle. All experimental procedures were performed according to standard work guidelines with due approval (Approval No. GWNU-2017-18) obtained from the Institutional Animal Care and Use Committee of the Gangneung-Wonju National University.

In this study, gerbils (total *n* = *63*) were used. The experimental groups were as follows. There are the Sham group (*n* = *7*) and Induction of transient cerebral ischemia group (Isch group; Ischemia group) (*n* = *7*). The experimental group corresponding to the application time of cathodal tDCS after induction of transient cerebral ischemia (at 0, 5, 10, and 60 min) is divided into 0, 5, 10, and 60 min group (*n* = *7* per group). The experimental group corresponding to the persistence of cathodal tDCS after induction of transient cerebral ischemia (after 5, 7, and 10 days)) is divided into 5,7 and 10 days group (*n* = *7* per group).

### Induction of transient cerebral ischemia in Mongolian gerbils

Prior to undergoing surgery, the experimental animals were anesthetized with 2.5% isoflurane (HANA PHARM CO., Seoul, Korea) mixed with 34% oxygen and 66% nitrous oxide. A rectal thermometer (TR-100, YSU, USA), along with a heating pad maintained a stable temperature of 37 ± 0.3 °C, was used during the surgery. After exposing the common carotid artery of the experimental animals, it was retracted using 4-0 silk (AILEE, Busan, Korea). Subsequently, the common carotid artery was subjected to clip ligation for 5 min in order to occlude the blood flow. After 5 min, the aneurysm clip was removed, and reperfusion of blood flow was examined. Following the induction of ischemic stroke, the experimental animals were maintained at body temperature in a thermostat to facilitate their recovery. The sham group underwent the same surgical procedure without clip ligation of the common carotid artery. Induction of ischemia was according to the protocol by O'Neill et al. [13].

### Electrode fixture implantation and application

**Fig. 5:**
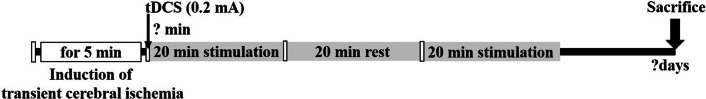
Protocol of cathodal tDCS application after induction of transient cerebral ischemia. After induction of transient cerebral ischemia for 5 min, “20 min stimulation—20 min rest—20 min stimulation” protocol applied in this experiment

Experimental animals were anesthetized with 2.5% isoflurane mixed with 34% oxygen and 66% nitrous oxide. A rectal thermometer (TR-100), along with a heating pad maintained a stable temperature of 37 ± 0.3 °C, was used during electrode implantation and application. An active electrode fixture (cathode) having a size of 5 mm was placed in the calvaria where the bregma is located. while a contrast electrode fixture (positive electrode) was placed on the thoracic skin. Subsequently, a current stimulator was attached to the electrode fixture, and the “20 min stimulation-20 min rest-20 min stimulation” protocol was implemented for the application of cathodal tDCS [22, 23] (Fig. 5). All tDCS applications were performed according to this protocol.

### Application of cathodal tDCS

#### Application time of cathodal tDCS after induction of transient cerebral ischemia (at 0, 5, 10, and 60 min)

After induction of transient cerebral ischemia, cathodal tDCS was applied to confirm the alterations within the neurons in the hippocampal CA1 regions, in accordance with the timing of cathodal tDCS application. The application of cathodal tDCS was performed at an intensity of 0.2 mA immediately following the induction of transient cerebral ischemia (0 min), as well as after intervals of 5 min, 10 min, and 60 min. After 5 days, the brain tissue was extracted and analyzed histologically (Fig. 1A).

#### Persistence of cathodal tDCS after induction of transient cerebral ischemia (after 5, 7, and 10 days)

Cathodal tDCS was applied immediately after the induction of transient cerebral ischemia. To confirm the persistence of this tDCS, brain tissue was extracted 5 days, 7 days, and 10 days after its application. This was followed by histological analysis of the brain tissue (Fig. 3A).

### Tissue preparation

To perform histochemical analysis, the animals were first anesthetized. They were perfused intracardially with 0.9% saline and 4% paraformaldehyde in phosphate-buffered saline (pH 7.5). Brain tissues were subsequently extracted and post-fixed with the same fixative for 8 h. Thereafter, the brain tissues were treated with 30% sucrose solution at room temperature for 12 h. The treated tissues were cut to a thickness of 30 µm using a frozen section and then stored in a preservation solution at 4 °C for further studies.

### CV staining

Following the induction of transient cerebral ischemia, CV staining of the surrounding nuclei of surviving neurons was performed to confirm the neuronal protective effect according to the stimulation intensity of cathodal tDCS. Brain tissue was mounted on slides coated with gelatin, followed by staining with 1.0% (w/v) CV acetate solution for 40 min. Subsequently, the stained brain tissues were dehydrated in 70% to 100% ethanol and clarified using xylene. They were then mounted using Canada balsam (Kato, Japan). The completed slides were observed under a Axio Imager A2 microscope (Carl Zeiss, Oberkochen, Germany).

### F-J C staining

After induction of transient cerebral ischemia, neuronal cell death in the hippocampal CA1 region was confirmed via fluorescent staining of neurons using F-J C staining. The brain tissues were treated with basic alcohol for 5 min and washed with 70% alcohol and distilled water for 2 min. Subsequently, the brain tissues were treated with 0.06% potassium permanganate solution for 20 min and washed twice with distilled water for 2 min each. Staining of the tissues with 0.001% Fluoro-Jade C (Histochem, Jefferson, USA) solution (containing 0.1% acetic acid) was performed for 30 min. Thereafter, the stained brain tissues were washed thrice with distilled water for 1 min each. Finally, the stained brain tissues were dried for 60 min on a slide warmer and mounted using dibutylphthalate polystyrene xylene (DPX) (Sigma, St. Louis, MO, USA). The completed slides were observed under a Axio Imager A2 fluorescence microscope (Carl Zeiss, Oberkochen, Germany) at an excitation wavelength (Ex) of 385 nm and emission wavelength (Em) of 425 nm.

### Statistical analysis

The most predominant sites of the stained brain tissues were photographed using a microscope equipped with a CCD camera. To quantitatively analyze of surviving neuron and apoptosis, 10 section per animal were selected with 120 µm interval. The neurons were obtained in a 250 X 250 µm square at the same area of each subregion and counted by averaging total numbers using an image J analyzing system.

The numerical data obtained in this study were expressed as their mean ± SEM. Differences between the two groups were statistically analyzed using one-way ANOVA and Tukey post-hoc test. Data were considered statistically significant if the p-value was less than 0.05.

## Data Availability

All data produced and analyzed in the current study are included in this paper.

## Abbreviations

ANOVA: Analysis of variance
BBB: Blood–brain barrier
CA: Cornu ammonis
CCD: Charge-Coupled Device
CV: Cresyl violet staining
DCEFs: Direct current electric fields
DPX: Dibutylphthalate polystyrene xylene
F-J C: Fluoro-Jade C
Isch: Ischemia
MCA: Middle cerebral artery
NBO: Normobaric hyperoxia
NBP: Dl-3n-butylphthalide\
tDCS: Transcranial direct current stimulation
tPA: Tissue-type plasminogen activator

## References

[1] Rosamond W, Flegal K, Friday G, Furie K, Go A, Greenlund K (2007). Heart disease and stroke statistics–2007 update: a report from the American Heart Association Statistics Committee and Stroke Statistics Subcommittee. Circulation.

[2] Beal CC (2010). Gender and stroke symptoms: a review of the current literature. J Neurosci Nurs.

[3] Murphy TH, Li P, Betts K, Liu R (2008). Two-photon imaging of stroke onset in vivo reveals that NMDA-receptor independent ischemic depolarization is the major cause of rapid reversible damage to dendrites and spines. J Neurosci.

[4] Bretón RR, Rodríguez JCG (2012). Excitotoxicity and oxidative stress in acute ischemic stroke. Acute ischemic stroke Croatia/China.

[5] Ouyang YB, Voloboueva LA, Xu LJ, Giffard RG (2007). Selective dysfunction of hippocampal CA1 astrocytes contributes to delayed neuronal damage after transient forebrain ischemia. J Neurosci.

[6] Xu L, Emery JF, Ouyang YB, Voloboueva LA, Giffard RG (2010). Astrocyte targeted overexpression of Hsp72 or SOD2 reduces neuronal vulnerability to forebrain ischemia. Glia.

[7] Peruzzotti-Jametti L, Cambiaghi M, Bacigaluppi M, Gallizioli M, Gaude E, Mari S (2013). Safety and efficacy of transcranial direct current stimulation in acute experimental ischemic stroke. Stroke.

[8] Braun R, Klein R, Walter H, Ohren M, Freudenmacher L, Getachew K (2016). Transcranial direct current stimulation accelerates recovery of function, induces neurogenesis and recruits oligodendrocyte precursors in a rat model of stroke. Exp Neurol.

[9] Fregni F, Pascual-Leone A (2007). Technology insight: noninvasive brain stimulation in neurology-perspectives on the therapeutic potential of rTMS and tDCS. Nat Clin Pract Neurol.

[10] George MS, Aston-Jones G (2010). Noninvasive techniques for probing neurocircuitry and treating illness: vagus nerve stimulation (VNS), transcranial magnetic stimulation (TMS) and transcranial direct current stimulation (tDCS). Neuropsychopharmacology.

[11] Jonker ZD, Gaiser C, Tulen JH, Ribbers GM, Frens MA, Selles RW (2021). No effect of anodal tDCS on motor cortical excitability and no evidence for responders in a large double-blind placebo-controlled trial. Brain Stimul.

[12] Pulsinelli W (1992). Pathophysioligy of acute ischaemic stroke. Lancet.

[13] O'Neill MJ, Clemens JA. Rodent models of focal cerebral ischemia. Curr protoc Neurosci. 2001; Chapter 9: Unit 9.6. 10.1002/0471142301.ns0906s12 18428554

[14] Zhang KY, Rui G, Zhang JP, Guo L, An GZ, Lin JJ (2020). Cathodal tDCS exerts neuroprotective effect in rat brain after acute ischemic stroke. BMC Neurosci.

[15] Jiang T, Xu RX, Zhang AW, Di W, Xiao ZJ, Miao JY (2012). Effects of transcranial direct current stimulation on hemichannel pannexin-1 and neural plasticity in rat model of cerebral infarction. Neuroscience.

[16] von Bernhardi R, Eugenín-von Bernhardi L, Eugenín J (2017). What is neural plasticity?. Adv Exp Med Biol.

[17] Advani R, Naess H, Kurz MW (2017). The golden hour of acute ischemic stroke. Scand J Trauma Resusc Emerg Med.

[18] Notturno F, Pace M, Zappasodi F, Cam E, Bassetti CL, Uncini A (2014). Neuroprotective effect of cathodal transcranial direct current stimulation in a rat stroke model. J Neurol Sci.

[19] Liang J, Qi Z, Liu W, Wang P, Shi W, Dong W (2015). Normobaric hyperoxia slows blood–brain barrier damage and expands the therapeutic time window for tissue-type plasminogen activator treatment in cerebral ischemia. Stroke.

[20] Liao SJ, Lin JW, Pei Z, Liu CL, Zeng JS, Huang RX (2009). Enhanced angiogenesis with dl-3n-butylphthalide treatment after focal cerebral ischemia in RHRSP. Brain Res.

[21] Yang Y, Li Q, Ahmad F, Shuaib A (2000). Survival and histological evaluation of therapeutic window of post-ischemia treatment with magnesium sulfate in embolic stroke model of rat. Neurosci Lett.

[22] Liebetanz D, Fregni F, Monte-Silva KK, Oliveira MB, Amancio-dos-Santos A, Nitsche MA (2006). After-effects of transcranial direct current stimulation (tDCS) on cortical spreading depression. NeurosciLett.

[23] Liebetanz D, Koch R, Mayenfels S, König F, Paulus W, Nitsche MA (2009). Safety limits of cathodal transcranial direct current stimulation in rats. Clin Neurophysiol.

